# A New Family of Bivariate Exponential Distributions with Negative Dependence Based on Counter-Monotonic Shock Method

**DOI:** 10.3390/e23050548

**Published:** 2021-04-29

**Authors:** Rachid Bentoumi, Farid El Ktaibi, Mhamed Mesfioui

**Affiliations:** 1 Department of Mathematics and Statistics, Zayed University, Abu Dhabi 144534, United Arab Emirates; rachid.bentoumi@zu.ac.ae; 2 Département de Mathématiques et d’Informatiques, Université du Québec à Trois-Rivières, Trois-Rivières, QC G8Z 4M3, Canada; mhamed.mesfioui@uqtr.ca

**Keywords:** bivariate exponential distributions, common shock, counter-monotonic, dependence modeling, Fréchet bound, negative dependence

## Abstract

We introduce a new family of bivariate exponential distributions based on the counter-monotonic shock model. This family of distribution is easy to simulate and includes the Fréchet lower bound, which allows to span all degrees of negative dependence. The construction and distributional properties of the proposed bivariate distribution are presented along with an estimation of the parameters involved in our model based on the method of moments. A simulation study is carried out to evaluate the performance of the suggested estimators. An extension to the general model describing both negative and positive dependence is sketched in the last section of the paper.

## 1. Introduction

Exponential distributions are undoubtedly among the most popular and used distributions in many areas of application. They play a prominent role in a variety of fields, including reliability, hydrology, engineering, telecommunication, biological and environmental sciences, among others.

However, the exponential distribution cannot be naturally extended to the bivariate or the multivariate case in a unique way. As a result, the literature on bivariate exponential distributions is vast, including many different classes and models that have been developed in the past decades, for example, refs. [1,2,3,4,5,6,7,8,9,10], among others.

It is worth mentioning that most of the bivariate exponential models proposed in the literature are restricted to the case of non-negative dependence. Very few models have negative or both positive and negative correlation but do not fully complete the range of correlation $\left[1-\frac{\pi^{2}}{6},1\right]$ (see Moran [11]) and necessitate a complex structure in their construction.

The main aim of this paper is to present a new bivariate exponential model that fully covers the negative dependence. To that end, we will adopt a technique based on the counter-monotonic shock model, which was introduced by Genet et al. [12]. This procedure is quite different from the common shock method used by Marshal and Olkin [8] to define a family of bivariate exponential distributions. Indeed, the latter is limited to model the positive dependence and imposes restrictions on the correlation structure, especially when the marginal are not identically distributed. In contrast, the counter-monotonic shock technique provides a flexible framework for building negatively correlated bivariate exponential distributions. Thanks to an appropriate parametrization, the resulting model can be viewed as a family of bivariate exponential distributions with given marginals and can be provided with a dependence parameter inducing the dependence in the model. In addition, this family of distribution is easy to simulate and includes the Fréchet lower bound, which allows describing the full negative range of correlation, namely $\left[1-\frac{\pi^{2}}{6},0\right]$.

We proceed as follows: We introduce our novel class of bivariate exponential distributions based on the counter-monotonic shock model in the next section. The derivation of the probability density function of this distribution is given in Section 3. We also present in this section the joint moment generating function, monotonicity, singularity, and scaling properties. Estimation of the model parameters through the moment method is discussed in Section 4. The proposed framework will be illustrated by simulations in Section 5. Concluding comments and directions for further research are presented in Section 6.

## 2. The Model

In order to define the suggested bivariate distribution, let us first recall the notion of a counter-monotonic random pair. Let *K* be a joint distribution with given marginal distributions *F* and *G*. The next double inequalities are due to [13]:

$$\sup\left[F(x)+G(y)-1,0\right]\leq K\left(x,y\right)\leq \min\left[F(x),G(y)\right].$$

As pointed out by Fréchet, these bounds are themselves bivariate distributions with the same marginals *F* and *G*. The counter-monotonic concept is related to the lower Fréchet bound, and it is defined as follows.

**Definition** **1.** *The random pair (X,Y) with marginal distributions F and G, respectively, is counter-monotonic if its joint distribution function is the lower Fréchet bound. Equivalently, there exists a unit uniform random variable U such that $X=F^{-1}\left(U\right)$ and $Y=G^{-1}\left(1-U\right)$.*

Note that the counter-monotonic notion describes the perfect negative dependence.

### The New Bivariate Exponential Distribution

In the following, we introduce a new family of bivariate exponential distributions with given marginals describing the negative dependence. The idea is based on the counter-monotonic shock method introduced in [12]. The principle of this approach is to link independent exponential random variables through counter-monotonic ones in order to produce negative dependence. To this end, let $\lambda_{i}>0$, i=1,2 be the marginal parameters and let θ∈(0,1) denote the dependence parameter.

**Definition** **2.** *Let $\left(X_{1},X_{2}\right)$ and $\left(Y_{1},Y_{2}\right)$ be independent random pairs such that $Y_{i}$∼Exp$\left(\theta \lambda_{i}\right)$ and $X_{i}$∼Exp$\left(\lambda_{i}\left(1-\theta \right)\right)$, i=1,2. Denote by $G_{i}$ the distribution functions of* $Y_{i},\;i=1,\;2,$ *respectively. Suppose further that*

*1.*  *$Y_{1}$ and $Y_{2}$ are counter-monotonic, that is, $Y_{1}=G_{1}^{-1}\left(U\right)$ and $Y_{2}=G_{2}^{-1}\left(1-U\right)$, where U is uniformly distributed over [0,1].*
*2.*  *$X_{1},X_{2}$ and U are independent.*

*The distribution of the random pair (X,Y), defined by*

(1) $$\begin{array}{c} X=\min\left(X_{1},Y_{1}\right)\;and\;Y=\min\left(X_{2},Y_{2}\right), \end{array}$$

*is called the counter-monotonic shock bivariate exponential distribution.*

Note that the set of all random pairs defined by (1) will be denoted BED${}^{-}\left(\theta ,\Lambda \right)$, where $\Lambda =(\lambda_{1},\lambda_{2})$. Clearly, the latter is a family of bivariate exponential distributions with given marginals, since by construction, X∼Exp$\left(\lambda_{1}\right)$ and Y∼Exp$\left(\lambda_{2}\right)$. The parameter θ∈(0,1) does not affect the marginal distributions; it can be interpreted as a dependence parameter. In fact, one observes that this family reaches the independence case when θ goes to 0 and it approaches the perfect negative dependence described by the Fréchet lower bound when θ goes to 1, respectively.

Thanks to the relations $Y_{1}=G_{1}^{-1}\left(U\right)$ and $Y_{2}=G_{2}^{-1}\left(1-U\right)$, one deduces an interesting alternative representation of (1) given by

(2) $$\begin{array}{c} X=\min\left(X_{1},\frac{Z}{\lambda_{1}\theta }\right)\;\mathrm{and}\;Y=\min\left(X_{2},-\frac{\ln\left\{1-e^{-Z}\right\}}{\lambda_{2}\theta }\right) \end{array}$$

where *Z* is an exponential random variable, with parameter 1, which is independent of $X_{1}$ and $X_{2}$. This presentation provides an easy way to simulate data from this model through the next steps:

1. Consider $\lambda_{1}>0$, $\lambda_{2}>0$ and θ∈(0,1).
2. Generate independent values $x_{1}$, $x_{2}$ and *z* from $\mathrm{Exp}(\lambda_{1}\left(1-\theta \right))$, $\mathrm{Exp}(\lambda_{2}\left(1-\theta \right))$ and Exp(1), respectively.
3. Set $x=\min\left(x_{1},z/\lambda_{1}\theta \right)$ and $y=\min\left(x_{2},-\ln\left\{1-e^{-z}\right\}/\lambda_{2}\theta \right)$.
4. The desired pair is (x,y).

The following figure displays simulated data using the previous algorithm with θ=0.5, $\lambda_{1}=1$ and $\lambda_{2}=2$.

**Figure 1:**
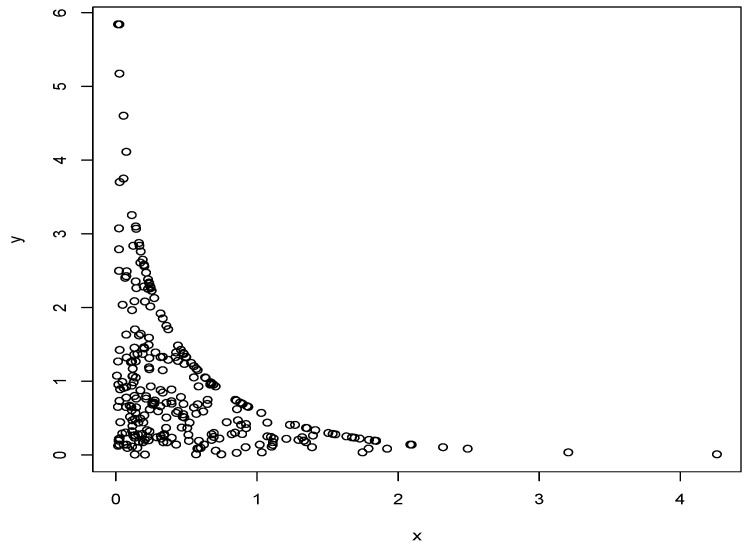
Simulated random pairs from BED${}^{-}\left(\theta ,\Lambda \right)$.

As illustrated in Figure 1, the proposed distribution seems to have both absolutely continuous and singular components. This interesting property will be examined in Proposition 2.

## 3. Properties of the New Bivariate Exponential Distribution

The following section will be consecrated to investigating the properties of the new family of bivariate exponential distribution based on the counter-monotonic shocks. We will start with the joint survival function of the distribution and then derive the corresponding joint probability density function. It will then be followed by an analysis of the product moment of the distribution, the coefficient of correlation, and the moment generating function. Monotonicity and scaling properties will also be discussed.

### 3.1. Survival and Density Functions

We now study the joint survival function associated with the new family BED${}^{-}\left(\theta ,\Lambda \right)$ and then deduce the corresponding joint probability density function.

**Proposition** **1.** *The survival function of (X,Y)∼${\mathrm{BED}}^{-}\left(\theta ,\Lambda \right)$ is given by*

(3) $${\bar{K}}_{\theta }\left(x,y\right)=e^{-\lambda_{1}\left(1-\theta \right)x}e^{-\lambda_{2}\left(1-\theta \right)y}{\left[e^{-\lambda_{1}\theta x}+e^{-\lambda_{2}\theta y}-1\right]}_{+},$$

*where $x_{+}=\max\left(x,0\right)$ for any x∈R.*

**Proof.**  Using the fact that the random variables $X_{1}$, $X_{2}$ and *U* are independent, one has from (1), for all $\left(x,y\right)\in R_{+}^{2}$,

$$\begin{array}{ccc} {\bar{K}}_{\theta }\left(x,y\right) & = & P(X\geq x,Y\geq y)\\ & = & P(X_{1}\geq x,X_{2}\geq y,G_{1}^{-1}\left(U\right)\geq x,G_{2}^{-1}\left(1-U\right)\geq y)\\ & = & P(X_{1}\geq x,X_{2}\geq y,G_{1}\left(x\right)\leq U\leq 1-G_{2}\left(y\right))\\ & = & P\left(X_{1}\geq x\right)P\left(X_{2}\geq y\right)P\left(G_{1}\left(x\right)\leq U\leq 1-G_{2}\left(y\right)\right)\\ & = & P\left(X_{1}\geq x\right)P\left(X_{2}\geq y\right){\left[1-G_{1}\left(x\right)-G_{2}\left(y\right)\right]}_{+}\\ & = & e^{-\lambda_{1}\left(1-\theta \right)x}e^{-\lambda_{2}\left(1-\theta \right)y}{\left[e^{-\lambda_{1}\theta x}+e^{-\lambda_{2}\theta y}-1\right]}_{+} \end{array}$$

which completes the proof of the proposition. □

**Corollary** **1.** *The joint distribution of (X,Y)∼${BED}^{-}\left(\theta ,\Lambda \right)$ is given by*

$$\begin{array}{c} K_{\theta }\left(x,y\right)=1-e^{-\lambda_{1}x}-e^{-\lambda_{2}y}+e^{-\lambda_{1}\left(1-\theta \right)x}e^{-\lambda_{2}\left(1-\theta \right)y}{\left[e^{-\lambda_{1}\theta x}+e^{-\lambda_{2}\theta y}-1\right]}_{+}, \end{array}$$

*where $x_{+}=\max\left(x,0\right)$ for any x∈R.*

**Proof.**  Using the relation between the $K_{\theta }$ and ${\bar{K}}_{\theta }$

$$K_{\theta }\left(x,y\right)={\bar{K}}_{\theta }\left(x,y\right)+F\left(x\right)+G\left(y\right)-1,$$

the result can be immediately deduced. □

Let us now recall the beta function and the incomplete beta function defined, respectively, by

$$\begin{array}{ccc} B(x,y) & = & \int_{0}^{1}t^{x-1}{\left(1-t\right)}^{y-1}dt,\;x,y>0,\\ B(x,a,b) & = & \int_{0}^{x}t^{a-1}{\left(1-t\right)}^{b-1}dt,\;a,b,x>0. \end{array}$$

These functions are linked to the beta distribution. In fact, a random variable *Z* follows a beta distribution with parameters a>0 and b>0 if its distribution is defined, for x∈[0,1], by

$$G_{a,b}\left(x\right)=\frac{B(x,a,b)}{B(a,b)}.$$

### 3.2. Singularity and Density Function

As shown below, the proposed family of bivariate exponential distribution possesses both an absolutely continuous and singular part involving the beta distribution. This property arises naturally in higher dimensions, as mentioned in [8].

**Proposition** **2.** *The survival function of (X,Y)∼${BED}^{-}\left(\theta ,\Lambda \right)$ is of the form*

(4) $$\begin{array}{c} {\bar{K}}_{\theta }\left(x,y\right)=B\left(\frac{1}{\theta },\frac{1}{\theta }\right){\bar{K}}_{s,\theta }\left(x,y\right)+\left(1-B\left(\frac{1}{\theta },\frac{1}{\theta }\right)\right){\bar{K}}_{c,\theta }\left(x,y\right), \end{array}$$

*where*

$${\bar{K}}_{s,\theta }\left(x,y\right)=\left\{\begin{array}{cc} G_{\frac{1}{\theta },\frac{1}{\theta }}\left(e^{-\lambda_{1}\theta x}\right)-G_{\frac{1}{\theta },\frac{1}{\theta }}\left(1-e^{-\lambda_{2}\theta y}\right) & \;if\;e^{-\lambda_{1}\theta x}+e^{-\lambda_{2}\theta y}-1>0,\;\\ 0 & \;if\;e^{-\lambda_{1}\theta x}+e^{-\lambda_{2}\theta y}-1\leq 0\; \end{array}\right.$$

*is a singular survival function, and*

$${\bar{K}}_{c,\theta }\left(x,y\right)=\frac{1}{1-B\left(\frac{1}{\theta },\frac{1}{\theta }\right)}{\bar{K}}_{\theta }\left(x,y\right)-\frac{B\left(\frac{1}{\theta },\frac{1}{\theta }\right)}{1-B\left(\frac{1}{\theta },\frac{1}{\theta }\right)}{\bar{K}}_{s,\theta }\left(x,y\right)$$

*is an absolutely continuous survival function.*

**Proof.**  Let $\left(u,v\right)\in {\left(0,\infty \right]}^{2}$ such that $e^{-\lambda_{1}\theta u}+e^{-\lambda_{2}\theta v}-1>0$. Using (3), we get

$$\begin{array}{ccc} \frac{\partial^{2}{\bar{K}}_{\theta }}{\partial u\partial v}\left(u,v\right) & = & \lambda_{1}\lambda_{2}\left(1-\theta \right)e^{-\lambda_{1}u}e^{-\lambda_{2}\left(1-\theta \right)v}+\lambda_{1}\lambda_{2}\left(1-\theta \right)e^{-\lambda_{1}\left(1-\theta \right)u}e^{-\lambda_{2}v}\\ & - & \lambda_{1}\lambda_{2}{\left(1-\theta \right)}^{2}e^{-\lambda_{1}\left(1-\theta \right)u}e^{-\lambda_{2}\left(1-\theta \right)v}. \end{array}$$

Define for x>0,

(5) $$b_{1}\left(x\right)=-\frac{\ln\left(1-e^{-\lambda_{2}\theta x}\right)}{\lambda_{1}\theta }\;\mathrm{and}\;b_{2}\left(x\right)=-\frac{\ln\left(1-e^{-\lambda_{1}\theta x}\right)}{\lambda_{2}\theta }.$$

It follows that if $e^{-\lambda_{1}\theta x}+e^{-\lambda_{2}\theta y}-1>0$,

$$\begin{array}{c} \int_{x}^{\infty }\int_{y}^{\infty }\frac{\partial^{2}{\bar{K}}_{\theta }}{\partial u\partial v}\left(u,v\right)dudv=\int_{x}^{b_{1}\left(y\right)}\left(\int_{y}^{b_{2}\left(u\right)}\frac{\partial^{2}{\bar{K}}_{\theta }}{\partial u\partial v}\left(u,v\right)dv\right)du\\ \;=\lambda_{1}\lambda_{2}\left(1-\theta \right)\int_{x}^{b_{1}\left(y\right)}e^{-\lambda_{1}u}\left(\int_{y}^{b_{2}\left(u\right)}e^{-\lambda_{2}\left(1-\theta \right)v}dv\right)du\\ \;+\lambda_{1}\lambda_{2}\left(1-\theta \right)\int_{x}^{b_{1}\left(y\right)}e^{-\lambda_{1}\left(1-\theta \right)u}\left(\int_{y}^{b_{2}\left(u\right)}e^{-\lambda_{2}v}dv\right)du\\ \;-\lambda_{1}\lambda_{2}{\left(1-\theta \right)}^{2}\int_{x}^{b_{1}\left(y\right)}e^{-\lambda_{1}\left(1-\theta \right)u}\left(\int_{y}^{b_{2}\left(u\right)}e^{-\lambda_{2}\left(1-\theta \right)v}dv\right)du\\ \;=J_{1}+J_{2}-J_{3}. \end{array}$$

It could be readily seen that

$$\begin{array}{ccc} J_{1} & = & e^{-\lambda_{2}\left(1-\theta \right)y}e^{-\lambda_{1}x}-e^{-\lambda_{2}\left(1-\theta \right)y}{\left(1-e^{-\lambda_{2}\theta y}\right)}^{\frac{1}{\theta }}\\ & & -\frac{1}{\theta }\left\{B\left(e^{-\lambda_{1}\theta x},\frac{1}{\theta },\frac{1}{\theta }\right)-B\left(1-e^{-\lambda_{2}\theta y},\frac{1}{\theta },\frac{1}{\theta }\right)\right\},\\ J_{2} & = & e^{-\lambda_{1}\left(1-\theta \right)x}e^{-\lambda_{2}y}-e^{-\lambda_{2}y}{\left(1-e^{-\lambda_{2}\theta y}\right)}^{\frac{1}{\theta }-1}\\ & & -\frac{1-\theta }{\theta }\left\{B\left(e^{-\lambda_{1}\theta x},\frac{1}{\theta }-1,\frac{1}{\theta }+1\right)-B\left(1-e^{-\lambda_{2}\theta y},\frac{1}{\theta }-1,\frac{1}{\theta }+1\right)\right\} \end{array}$$

and

$$\begin{array}{ccc} J_{3} & = & e^{-\lambda_{1}\left(1-\theta \right)x}e^{-\lambda_{2}\left(1-\theta \right)y}-e^{-\lambda_{2}\left(1-\theta \right)y}{\left(1-e^{-\lambda_{2}\theta y}\right)}^{\frac{1}{\theta }-1}\\ & & -\frac{1-\theta }{\theta }\left\{B\left(e^{-\lambda_{1}\theta x},\frac{1}{\theta }-1,\frac{1}{\theta }\right)-B\left(1-e^{-\lambda_{2}\theta y},\frac{1}{\theta }-1,\frac{1}{\theta }\right)\right\}.\;\;\; \end{array}$$

By virtue of the identity,

$$\begin{array}{c} B(x,a+1,b)+B(x,a,b+1)=B(x,a,b), \end{array}$$

the quantity $J_{1}+J_{2}-J_{3}$ reduces to

(6) $$\begin{array}{c} \int_{x}^{\infty }\int_{y}^{\infty }\frac{\partial^{2}{\bar{K}}_{\theta }}{\partial u\partial v}\left(u,v\right)dudv=\\ \;{\bar{K}}_{\theta }\left(x,y\right)-\left\{B\left(e^{-\lambda_{1}\theta x},\frac{1}{\theta },\frac{1}{\theta }\right)-B\left(1-e^{-\lambda_{2}\theta y},\frac{1}{\theta },\frac{1}{\theta }\right)\right\}. \end{array}$$

The singular component of the survival function is then given by

(7) $$\begin{array}{c} \alpha {\bar{K}}_{s}\left(x,y\right)=B\left(e^{-\lambda_{1}\theta x},\frac{1}{\theta },\frac{1}{\theta }\right)-B\left(1-e^{-\lambda_{2}\theta y},\frac{1}{\theta },\frac{1}{\theta }\right) \end{array}$$

where the normalized constant α is obtained by tending (x,y) to (0,0) in the previous formula. This leads to $\alpha =B\left(\frac{1}{\theta },\frac{1}{\theta }\right)$.

Similarly, the continuous part of the survival function is given by

(8) $$\begin{array}{c} {\bar{K}}_{c}\left(x,y\right)=\left\{1-B\left(\frac{1}{\theta },\frac{1}{\theta }\right)\right\}\int_{x}^{\infty }\int_{y}^{\infty }\frac{\partial^{2}{\bar{K}}_{\theta }}{\partial u\partial v}\left(u,v\right)dudv. \end{array}$$

Putting (6)–(8) together, we have the desired decomposition. This ends the proof of Proposition 2. □

The density function of the proposed family of distribution can be obtained immediately from the previous result.

**Corollary** **2.** *The density function of (X,Y)∼${BED}^{-}\left(\theta ,\Lambda \right)$ is expressed by,*

$$\begin{array}{c} f_{\theta }\left(x,y\right)=\left\{\begin{array}{cc} f_{1}\left(x,y\right) & \;if\;e^{-\lambda_{1}\theta x}+e^{-\lambda_{2}\theta y}-1>0,\;\\ f_{0}\left(x\right) & \;\;in\;the\;curve\;\left\{\left(x,y\right)\in R_{+}^{2}:e^{-\lambda_{1}\theta x}+e^{-\lambda_{2}\theta y}-1=0\right\}, \end{array}\right. \end{array}$$

*where*

(9) $$\begin{array}{ccc} f_{1}\left(x,y\right) & = & \frac{\partial^{2}{\bar{K}}_{\theta }}{\partial x\partial y}\left(x,y\right)\\ & = & \lambda_{1}\lambda_{2}\left(1-\theta \right)e^{-\lambda_{1}\left(1-\theta \right)x}e^{-\lambda_{2}\left(1-\theta \right)y}\left(e^{-\lambda_{1}\theta x}+e^{-\lambda_{2}\theta y}-1+\theta \right) \end{array}$$

*and*

$$f_{0}\left(x\right)=\lambda_{1}\theta e^{-\lambda_{1}x}{\left(1-e^{-\lambda_{1}\theta x}\right)}^{\frac{1}{\theta }-1}.$$

**Proof.**  It is easily seen that $f_{1}$ is the density function corresponding to the continuous part of the survival function ${\bar{K}}_{s,\theta }$ described by (9).

Next, from Proposition 2, the singular component of the survival function of (X,Y) is given by

$$P\left(X>x,Y>y\right)={\bar{K}}_{s,\theta }\left(x,y\right)=G_{\frac{1}{\theta },\frac{1}{\theta }}\left(e^{-\lambda_{1}\theta x}\right)-G_{\frac{1}{\theta },\frac{1}{\theta }}\left(1-e^{-\lambda_{2}\theta y}\right).$$

This ensures that the survival function of *X* in the singular part is ${\bar{F}}_{0}\left(x\right)=P\left(X>x\right)=G_{\frac{1}{\theta },\frac{1}{\theta }}\left(e^{-\lambda_{1}\theta x}\right)$. Consequently, the density function in the curve $A=\{\left(x,y\right)\in R_{+}^{2}:e^{-\lambda_{1}\theta x}+e^{-\lambda_{2}\theta y}-1=0\}$ is

$$f_{0}\left(x\right)=-{\bar{F}}_{0}^{\prime }\left(x\right)=\lambda_{1}\theta e^{-\lambda_{1}\theta x}G_{\frac{1}{\theta },\frac{1}{\theta }}^{\prime }\left(e^{-\lambda_{1}\theta x}\right)=\lambda_{1}\theta e^{-\lambda_{1}x}{\left(1-e^{-\lambda_{1}\theta x}\right)}^{\frac{1}{\theta }-1}.$$

This ends the proof of Corollary 2. □

One can check that

$$\int_{0}^{\infty }\int_{0}^{\infty }f_{\theta }\left(x,y\right)dxdy=\int_{e^{-\lambda_{1}\theta x}+e^{-\lambda_{2}\theta y}-1>0}f_{1}\left(x,y\right)dxdy+\int_{0}^{\infty }f_{0}\left(x\right)dx=1$$

which guarantees that $f_{\theta }$ is a density function. In addition, one observes that the probability that (X,Y) lies in the singular part represented by the curve $A=\{\left(x,y\right)\in R_{+}^{2}:e^{-\lambda_{1}\theta x}+e^{-\lambda_{2}\theta y}-1=0\}$ is given by:

$$\begin{array}{ccc} \int_{0}^{\infty }f_{0}\left(x\right)dx & = & \int_{0}^{\infty }\lambda_{1}\theta e^{-\lambda_{1}x}{\left(1-e^{-\lambda_{1}\theta x}\right)}^{\frac{1}{\theta }-1}dx\\ & = & \int_{0}^{1}u^{\frac{1}{\theta }-1}{\left(1-u\right)}^{\frac{1}{\theta }-1}du\\ & = & B\left(\frac{1}{\theta },\frac{1}{\theta }\right). \end{array}$$

### 3.3. Monotonicity

In the following, we show that the family ${\mathrm{BED}}^{-}\left(\theta ,\Lambda \right)$ is ordered in terms of θ in the negative quadrant dependence ordering. This means that the parameter θ can be considered as a dependence parameter for the family ${\mathrm{BED}}^{-}\left(\theta ,\Lambda \right)$.

**Proposition** **3.** *For $\left(\theta_{1},\theta_{2}\right)\in {\left[0,1\right]}^{2}$, one has*

$$\theta_{1}\leq \theta_{2}\;⟹\;{\bar{K}}_{\theta_{1}}\left(x,y\right)\geq {\bar{K}}_{\theta_{2}}\left(x,y\right)\;\forall \left(x,y\right)\in R^{2}.$$

**Proof.**  We remark that the survival function can be rewritten as

$$\begin{array}{ccc} {\bar{K}}_{\theta }\left(x,y\right) & = & e^{-\lambda_{1}x}e^{-\lambda_{2}y}{\left[e^{\lambda_{1}\theta x}+e^{\lambda_{2}\theta y}-e^{\lambda_{1}\theta x}e^{\lambda_{2}\theta y}\right]}_{+}\\ & = & e^{-\lambda_{1}x}e^{-\lambda_{2}y}{\left[1-\left(e^{\lambda_{1}\theta x}-1\right)\left(e^{\lambda_{2}\theta y}-1\right)\right]}_{+}. \end{array}$$

Hence, the result is in force since $\theta ⟼1-\left(e^{\lambda_{1}\theta x}-1\right)\left(e^{\lambda_{2}\theta y}-1\right))$ is a decreasing function in θ∈[0,1] for all $\left(x,y\right)\in R_{+}^{2}$ and $u⟼u_{+}$ is an increasing function in u∈R. □

The above result shows that the strength of the dependence of the pair (X,Y) in BED${}^{-}\left(\theta ,\Lambda \right)$ decreases with θ∈[0,1]. Hence, the covariance as well as the correlation of random pairs in ${\mathrm{BED}}^{-}\left(\theta ,\Lambda \right)$ decrease with respect to θ. In addition, one sees that, for all $\left(x,y\right)\in R^{2}$,

$${\bar{K}}_{\theta }\left(x,y\right)\leq {\bar{K}}_{0}\left(x,y\right)=\bar{F}\left(x\right)\bar{G}\left(x\right).$$

The latter outlines that the components of $\left(X,Y\right)\in {\mathrm{BED}}^{-}\left(\theta ,\Lambda \right)$ are negatively quadrant dependent. In particular, the correlation of any $\left(X,Y\right)\in {\mathrm{BED}}^{-}\left(\theta ,\Lambda \right)$ is negative.

### 3.4. Product Moment and Correlation Structure

Recall the partial derivatives of the Beta function for x>0 and y>0

$$B_{p,q}\left(x,y\right)=\frac{\partial^{p+q}B}{\partial x^{p}\partial y^{q}}\left(x,y\right)=\int_{0}^{1}t^{x-1}{\left(1-t\right)}^{y-1}\ln^{p}\left(t\right)\ln^{q}\left(1-t\right)dt.$$

**Proposition** **4.** *For θ∈(0,1), the product moment of (X,Y)∼${BED}^{-}\left(\theta ,\Lambda \right)$ is given, for any positive integers i and j, by*

(10) $$\begin{array}{c} E\left(X^{i}Y^{j}\right)=\frac{i!j!}{\lambda_{1}^{i}\lambda_{2}^{j}{\left(1-\theta \right)}^{j}}+\frac{i!j!}{\lambda_{1}^{i}\lambda_{2}^{j}{\left(1-\theta \right)}^{i}}-\frac{i!j!}{\lambda_{1}^{i}\lambda_{2}^{j}{\left(1-\theta \right)}^{i+j}}\\ \;-\frac{i!j}{\lambda_{1}^{i}\lambda_{2}^{j}}\sum_{s=0}^{i-1}\frac{{\left(-1\right)}^{s+j+1}}{\theta^{j+s}s!}B_{j-1,s}\left(\frac{1}{\theta }-1,\frac{1}{\theta }+1\right)\\ \;-\frac{ij!}{\lambda_{1}^{i}\lambda_{2}^{j}}\sum_{s=0}^{j-1}\frac{{\left(-1\right)}^{s+i+1}}{\theta^{i+s}s!}B_{i-1,s}\left(\frac{1}{\theta }-1,\frac{1}{\theta }+1\right)\\ \;+\frac{i!j}{\lambda_{1}^{i}\lambda_{2}^{j}{\left(1-\theta \right)}^{i}}\sum_{s=0}^{i-1}\frac{{\left(1-\theta \right)}^{s}{\left(-1\right)}^{s+j+1}}{s!\theta^{s+j}}B_{j-1,s}\left(\frac{1}{\theta }-1,\frac{1}{\theta }\right). \end{array}$$

**Proof.**  Set $A=\{\left(x,y\right)\in R_{+}^{2}:e^{-\theta x}+e^{-\theta y}-1>0\}$, one can observe that

$$\begin{array}{ccc} E\left(X^{i}Y^{j}\right) & = & \int_{0}^{\infty }\;\;\;\int_{0}^{\infty }ijx^{i-1}y^{j-1}{\bar{K}}_{\theta }\left(x,y\right)dxdy\\ & = & \frac{ij}{\lambda_{1}^{i}\lambda_{2}^{j}}\int_{0}^{\infty }\;\;\;\int_{0}^{\infty }x^{i-1}y^{j-1}e^{-(1-\theta )x}e^{-(1-\theta )y}{\left[e^{-\theta x}+e^{-\theta y}-1\right]}_{+}dxdy\\ & = & \frac{ij}{\lambda_{1}^{i}\lambda_{2}^{j}}\int \;\;\;\int_{A}x^{i-1}y^{j-1}e^{-x}e^{-(1-\theta )y}dxdy\\ & & +\frac{ij}{\lambda_{1}^{i}\lambda_{2}^{j}}\int \;\;\;\int_{A}x^{i-1}y^{j-1}e^{-(1-\theta )x}e^{-y}dxdy\\ & & -\frac{ij}{\lambda_{1}^{i}\lambda_{2}^{j}}\int \;\;\;\int_{A}x^{i-1}y^{j-1}e^{-(1-\theta )x}e^{-(1-\theta )y}dxdy\\ & = & I_{1}+I_{2}-I_{3}. \end{array}$$

Define $a\left(x\right)=-\theta^{-1}\ln\left(1-e^{-\theta x}\right)$. Hence,

$$\begin{array}{ccc} I_{1} & = & \frac{ij}{\lambda_{1}^{i}\lambda_{2}^{j}}\int_{0}^{\infty }y^{j-1}e^{-(1-\theta )y}\left(\int_{0}^{a(y)}x^{i-1}e^{-x}dx\right)dy\\ & = & \frac{i!j}{\lambda_{1}^{i}\lambda_{2}^{j}}\int_{0}^{\infty }y^{j-1}e^{-(1-\theta )y}\left(1-e^{-a(y)}\sum_{s=0}^{i-1}\frac{a{\left(y\right)}^{s}}{s!}\right)dy\\ & = & \frac{i!j!}{\lambda_{1}^{i}\lambda_{2}^{j}{\left(1-\theta \right)}^{j}}-\frac{i!j}{\lambda_{1}^{i}\lambda_{2}^{j}}\sum_{s=0}^{i-1}\frac{1}{s!}\int_{0}^{\infty }y^{j-1}e^{-(1-\theta )y}e^{-a(y)}a{\left(y\right)}^{s}dy\\ & = & \frac{i!j!}{\lambda_{1}^{i}\lambda_{2}^{j}{\left(1-\theta \right)}^{j}}-\frac{i!j}{\lambda_{1}^{i}\lambda_{2}^{j}}\sum_{s=0}^{i-1}\frac{{\left(-1\right)}^{s+j+1}}{\theta^{j+s}s!}\int_{0}^{1}u^{\frac{1}{\theta }-2}{\left(1-u\right)}^{\frac{1}{\theta }}{\left[\ln\left(u\right)\right]}^{j-1}{\left[\ln\left(1-u\right)\right]}^{s}du\\ & = & \frac{i!j!}{\lambda_{1}^{i}\lambda_{2}^{j}{\left(1-\theta \right)}^{j}}-\frac{i!j}{\lambda_{1}^{i}\lambda_{2}^{j}}\sum_{s=0}^{i-1}\frac{{\left(-1\right)}^{s+j+1}}{\theta^{j+s}s!}B_{j-1,s}\left(\frac{1}{\theta }-1,\frac{1}{\theta }+1\right), \end{array}$$

where the second line follows from the fact that

$$\int_{0}^{t}y^{j-1}e^{-y}dy=\left(j-1\right)!\left(1-e^{-t}\sum_{s=0}^{j-1}\frac{t^{s}}{s!}\right).$$

Similarly, one has

$$\begin{array}{ccc} I_{2} & = & \frac{i!j!}{\lambda_{1}^{i}\lambda_{2}^{j}{\left(1-\theta \right)}^{i}}-\frac{ij!}{\lambda_{1}^{i}\lambda_{2}^{j}}\sum_{s=0}^{j-1}\frac{{\left(-1\right)}^{s+i+1}}{\theta^{i+s}s!}B_{i-1,s}\left(\frac{1}{\theta }-1,\frac{1}{\theta }+1\right). \end{array}$$

Finally, we have

$$\begin{array}{ccc} I_{3} & = & \frac{ij}{\lambda_{1}^{i}\lambda_{2}^{j}}\int \int_{A}x^{i-1}y^{j-1}e^{-(1-\theta )x}e^{-(1-\theta )y}dxdy\\ & = & \frac{ij}{\lambda_{1}^{i}\lambda_{2}^{j}{\left(1-\theta \right)}^{i}}\int_{0}^{\infty }y^{j-1}e^{-(1-\theta )y}\left(\int_{0}^{\left(1-\theta )a(y\right)}z^{i-1}e^{-z}dz\right)dx\\ & = & \frac{i!j}{\lambda_{1}^{i}\lambda_{2}^{j}{\left(1-\theta \right)}^{i}}\int_{0}^{\infty }y^{j-1}e^{-(1-\theta )y}\left(1-e^{-(1-\theta )a(y)}\sum_{s=0}^{i-1}\frac{{\left(\left(1-\theta \right)a\left(y\right)\right)}^{s}}{s!}\right)dy\\ & = & \frac{i!j!}{\lambda_{1}^{i}\lambda_{2}^{j}{\left(1-\theta \right)}^{i+j}}\\ & & -\frac{i!j}{\lambda_{1}^{i}\lambda_{2}^{j}{\left(1-\theta \right)}^{i}}\sum_{s=0}^{i-1}\frac{1}{s!}\int_{0}^{\infty }y^{j-1}e^{-(1-\theta )y}e^{-(1-\theta )a(y)}{\left(\left(1-\theta \right)a\left(y\right)\right)}^{s}dy\\ & = & \frac{i!j!}{\lambda_{1}^{i}\lambda_{2}^{j}{\left(1-\theta \right)}^{i+j}}-\frac{i!j}{\lambda_{1}^{i}\lambda_{2}^{j}{\left(1-\theta \right)}^{i}}\sum_{s=0}^{i-1}\frac{{\left(1-\theta \right)}^{s}{\left(-1\right)}^{s+j+1}}{s!\theta^{s+j}}B_{j-1,s}\left(\frac{1}{\theta }-1,\frac{1}{\theta }\right). \end{array}$$

This completes the proof of Proposition 4. □

Next, we derive the correlation coefficient of (X,Y)∼${\mathrm{BED}}^{-}\left(\theta ,\Lambda \right)$.

**Corollary** **3.** *The correlation coefficient of (X,Y)∼${BED}^{-}\left(\theta ,\Lambda \right)$ is given by*

$$\begin{array}{ccc} corr\left(X,Y\right) & = & \frac{1}{{\left(1-\theta \right)}^{2}}\left[B\left(\frac{1}{\theta },\frac{1}{\theta }\right)-\theta^{2}\right]. \end{array}$$

**Proof.**  Using Equation (10), one has for i=j=1,

(11) $$\begin{array}{ccc} E\left(XY\right) & = & \frac{2}{\lambda_{1}\lambda_{2}\left(1-\theta \right)}-\frac{1}{\lambda_{1}\lambda_{2}{\left(1-\theta \right)}^{2}}\\ & & -\frac{2}{\lambda_{1}\lambda_{2}\theta }B\left(\frac{1}{\theta }-1,\frac{1}{\theta }+1\right)+\frac{1}{\lambda_{1}\lambda_{2}\theta \left(1-\theta \right)}B\left(\frac{1}{\theta }-1,\frac{1}{\theta }\right). \end{array}$$

To complete the proof of the corollary, we will make use of the following properties of the beta function

$$B\left(x+1,y\right)=\frac{x}{x+y}B\left(x,y\right)\;\mathrm{and}\;B\left(x,y+1\right)=\frac{y}{x+y}B\left(x,y\right).$$

It results that

(12) $$\begin{array}{ccc} B\left(\frac{1}{\theta }-1,\frac{1}{\theta }\right) & = & \frac{2-\theta }{1-\theta }B\left(\frac{1}{\theta },\frac{1}{\theta }\right),\\ B\left(\frac{1}{\theta }-1,\frac{1}{\theta }+1\right) & = & \frac{1}{1-\theta }B\left(\frac{1}{\theta },\frac{1}{\theta }\right). \end{array}$$

Substituting (12) in (11), we get the following expression for the covariance of (X,Y),

(13) $$\begin{array}{c} \mathrm{cov}\left(X,Y\right)=E\left(XY\right)-\frac{1}{\lambda_{1}\lambda_{2}}=\frac{1}{\lambda_{1}\lambda_{2}{\left(1-\theta \right)}^{2}}\left\{B\left(\frac{1}{\theta },\frac{1}{\theta }\right)-\theta^{2}\right\}. \end{array}$$

The result follows by using the fact that $\mathrm{corr}\left(X,Y\right)=\lambda_{1}\lambda_{2}\mathrm{cov}\left(X,Y\right)$. □

Let Γ(F,G) be the space of all bivariate random variables with given exponential marginal distributions *F* and *G* with parameters $\lambda_{1}$ and $\lambda_{2}$, respectively. The minimal correlation in the space Γ(F,G) is calculated by using the lower Fréchet bound. This means that, for all (X,Y)∈Γ(F,G), one has,

$$\rho_{\min}=\mathrm{corr}\left(F^{-1}\left(U\right),G^{-1}\left(1-U\right)\right)\leq \mathrm{corr}\left(X,Y\right),$$

where *U* is a random variable uniformly distributed over [0,1]. The above minimal correlation is given by

$$\rho_{\min}=E\left[\ln\left(U\right)\ln\left(1-U\right)\right]-1=1-\frac{\pi^{2}}{6}.$$

Hence, the full correlation range of negative dependence in the space Γ(F,G) is $\left[1-\pi^{2}/6,0\right]$. Note the proposed family of distribution ${\mathrm{BED}}^{-}\left(\theta ,\Lambda \right)$ describes this full negative range of correlation since it includes the Fréchet lower bound. In particular, one has

$$\lim_{\theta \to 1}\left\{\frac{1}{{\left(1-\theta \right)}^{2}}\left[B\left(\frac{1}{\theta },\frac{1}{\theta }\right)-\theta^{2}\right]\right\}=\rho_{\min}=1-\frac{\pi^{2}}{6}\approx -0.6449341.$$

**Figure 2:**
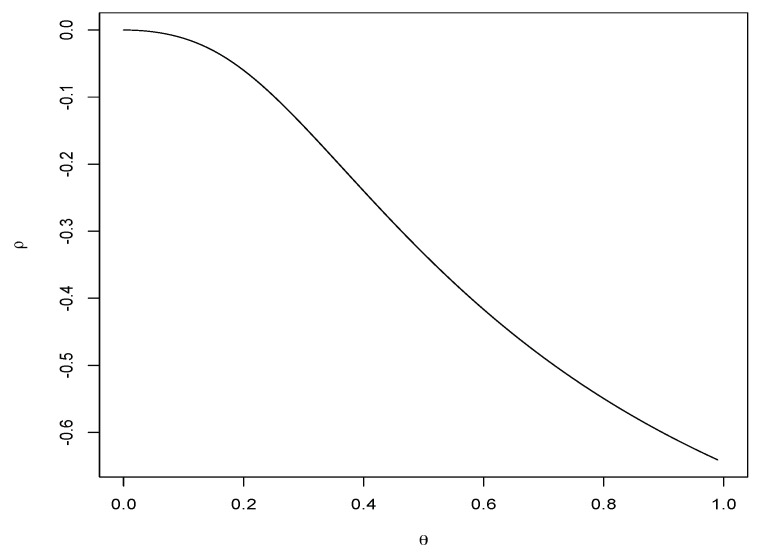
Coefficient of correlation in terms of the dependence parameter.

Figure 2 illustrates the behavior of the correlation coefficient in terms of the dependence parameter θ∈[0,1]. Note that the expression of the covariance described in (13) will be useful to estimate the dependence parameter using the method of moments.

### 3.5. Scaling Property

Analogously to the univariate case, the proposed family of distributions enjoys the scaling property.

**Proposition** **5.** *For any $a=\left(a_{1},a_{2}\right)\in \left(0,\infty \right)\times \left(0,\infty \right)$, one has*

$$\left(X,Y\right)∼{BED}^{-}\left(\theta ,\Lambda \right)\Leftrightarrow \left(a_{1}X,a_{2}Y\right)∼{BED}^{-}\left(\theta ,a^{-1}\Lambda \right),$$

*where $a^{-1}\Lambda =\left(\lambda_{1}/a_{1},\lambda_{2}/a_{2}\right)$.*

**Proof.**  The proof is straightforward, and therefore, it is omitted. □

### 3.6. Moment Generating Function

Here, we derive an explicit expression for the moment generating function of the family (X,Y)∼${\mathrm{BED}}^{-}\left(\theta ,\Lambda \right)$ in terms of the beta function.

**Proposition** **6.** *The moment generating function of (X,Y)∼${BED}^{-}\left(\theta ,\Lambda \right)$ is given, for all $\left(t,s\right)\in \left[0,\lambda_{1}\right]\times \left[0,\lambda_{2}\right]$, by*

$$\psi \left(t,s\right)=\frac{\lambda_{1}\lambda_{2}\left(1-\theta \right)}{\left(t-\lambda_{1}\left(1-\theta \right)\right)\left(s-\lambda_{2}\left(1-\theta \right)\right)}\left[\alpha_{1}B\left(\frac{\lambda_{1}-t}{\lambda_{1}\theta },\frac{\lambda_{2}-s}{\lambda_{2}\theta }\right)-\alpha_{2}+1\right],$$

*where $\alpha_{1}=\frac{st}{\lambda_{1}\lambda_{2}\left(1-\theta \right)}$ and $\alpha_{2}=\frac{\theta (\lambda_{1}\lambda_{2}-ts)}{\left(\lambda_{1}-t\right)\left(\lambda_{2}-s\right)}$*

**Proof.**  For the sake of easy reference, we restate $b_{1}\left(x\right)$ and $b_{2}\left(x\right)$ introduced earlier in (5)

$$b_{1}\left(x\right)=-\frac{\ln\left(1-e^{-\lambda_{2}\theta x}\right)}{\lambda_{1}\theta }\;\mathrm{and}\;b_{2}\left(x\right)=-\frac{\ln\left(1-e^{-\lambda_{1}\theta x}\right)}{\lambda_{2}\theta }.$$

Set $A_{1}=\left\{\left(x,y\right)\in R_{+}^{2}:e^{-\lambda_{1}\theta x}+e^{-\lambda_{2}\theta y}-1>0\right\}$ and $A_{2}=\left\{\left(x,y\right)\in R_{+}^{2}:e^{-\lambda_{1}\theta x}+e^{-\lambda_{2}\theta y}-1=0\right\}$. Hence,

(14) $$\begin{array}{ccc} \psi (t,s) & = & E\left(e^{tX+sY}\right)\\ & = & \int_{A_{1}}e^{tx+sy}f_{\theta }\left(x,y\right)dxdy+\int_{A_{2}}e^{tx+sy}f_{\theta }\left(x,y\right)dx\\ & = & C_{1}+C_{2}-C_{3}+C_{4} \end{array}$$

where

$$\begin{array}{ccc} C_{1} & = & \lambda_{1}\lambda_{2}\left(1-\theta \right)\int_{0}^{\infty }e^{(t-\lambda_{1})x}\left(\int_{0}^{b_{2}\left(x\right)}e^{(s-\lambda_{2}\left(1-\theta \right))y}dy\right)dx \end{array}$$

(15) $$\begin{array}{ccc} & = & \frac{\lambda_{2}\left(1-\theta \right)}{\theta \left(s-\lambda_{2}\left(1-\theta \right)\right)}B\left(\frac{\lambda_{1}-t}{\lambda_{1}\theta },\frac{\lambda_{2}-s}{\lambda_{2}\theta }\right)\\ & & -\frac{\lambda_{1}\lambda_{2}\left(1-\theta \right)}{\left(\lambda_{1}-t\right)\left(s-\lambda_{2}\left(1-\theta \right)\right)},\\ C_{2} & = & \lambda_{1}\lambda_{2}\left(1-\theta \right)\int_{0}^{\infty }e^{(s-\lambda_{2})y}\left(\int_{0}^{b_{1}\left(y\right)}e^{(t-\lambda_{1}\left(1-\theta \right))x}dx\right)dy \end{array}$$

(16) $$\begin{array}{ccc} & = & \frac{\lambda_{1}\left(1-\theta \right)}{\theta \left(t-\lambda_{1}\left(1-\theta \right)\right)}B\left(\frac{\lambda_{1}-t}{\lambda_{1}\theta },\frac{\lambda_{2}-s}{\lambda_{2}\theta }\right)\\ & & -\frac{\lambda_{1}\lambda_{2}\left(1-\theta \right)}{\left(\lambda_{2}-s\right)\left(t-\lambda_{1}\left(1-\theta \right)\right)},\\ C_{3} & = & \lambda_{1}\lambda_{2}{\left(1-\theta \right)}^{2}\int_{0}^{\infty }e^{(t-\lambda_{1}\left(1-\theta \right))x}\left(\int_{0}^{b_{2}\left(x\right)}e^{(s-\lambda_{2}\left(1-\theta \right))y}dy\right)dx\\ & = & \frac{\lambda_{2}{\left(1-\theta \right)}^{2}}{\theta \left(s-\lambda_{2}\left(1-\theta \right)\right)}B\left(\frac{\lambda_{1}-t}{\lambda_{1}\theta }-1,\frac{\lambda_{2}-s}{\lambda_{2}\theta }\right)\\ & & +\frac{\lambda_{1}\lambda_{2}{\left(1-\theta \right)}^{2}}{\left(t-\lambda_{1}\left(1-\theta \right)\right)\left(s-\lambda_{2}\left(1-\theta \right)\right)}. \end{array}$$

Using the fact that

$$B\left(x-1,y\right)=\frac{x-1+y}{x-1}B\left(x,y\right),$$

it follows that

$$\begin{array}{c} B\left(\frac{\lambda_{1}-t}{\lambda_{1}\theta }-1,\frac{\lambda_{2}-s}{\lambda_{2}\theta }\right)=\frac{\lambda_{1}s+\lambda_{2}t-\left(2-\theta \right)\lambda_{1}\lambda_{2}}{\lambda_{2}\left[t-\lambda_{1}\left(1-\theta \right)\right]}B\left(\frac{\lambda_{1}-t}{\lambda_{1}\theta },\frac{\lambda_{2}-s}{\lambda_{2}\theta }\right). \end{array}$$

Consequently, one gets

(17) $$\begin{array}{ccc} C_{3} & = & \frac{{\left(1-\theta \right)}^{2}\left[\lambda_{1}s+\lambda_{2}t-\left(2-\theta \right)\lambda_{1}\lambda_{2}\right]}{\theta \left(s-\lambda_{2}\left(1-\theta \right)\right)\left(t-\lambda_{1}\left(1-\theta \right)\right)}B\left(\frac{\lambda_{1}-t}{\lambda_{1}\theta },\frac{\lambda_{2}-s}{\lambda_{2}\theta }\right)\\ & & +\frac{\lambda_{1}\lambda_{2}{\left(1-\theta \right)}^{2}}{\left(t-\lambda_{1}\left(1-\theta \right)\right)\left(s-\lambda_{2}\left(1-\theta \right)\right)}. \end{array}$$

Finally,

(18) $$C_{4}=\lambda_{1}\theta \int_{0}^{\infty }e^{\left(t-\lambda_{1}\right)x+sb_{2}\left(x\right)}{\left(1-e^{-\lambda_{1}\theta x}\right)}^{\frac{1}{\theta }-1}dx=B\left(\frac{\lambda_{1}-t}{\lambda_{1}\theta },\frac{\lambda_{2}-s}{\lambda_{2}\theta }\right).$$

The result follows by inserting (15)–(18) in (14). □

## 4. Parameters Estimation

In the following, we estimate the parameters of the model $\Lambda =(\lambda_{1},\lambda_{2})$ in ${\left(0,\infty \right)}^{2}$ and θ∈[0,1] using the method of moments. To this end, let $\left(X_{1},Y_{1}\right),\cdots ,\left(X_{n},Y_{n}\right)$ be mutually independent copies of (X,Y)∼${\mathrm{BED}}^{-}\left(\theta ,\Lambda \right)$ and denote

$$\bar{X}=n^{-1}\sum_{i=1}^{n}X_{i},\;\bar{Y}=n^{-1}\sum_{i=1}^{n}Y_{i}\;\mathrm{and}\;S_{12}=\frac{1}{n-1}\sum_{i=1}^{n}\left(X_{i}-\bar{X}\right)\left(Y_{i}-\bar{Y}\right).$$

The sample means $\bar{X}$ and $\bar{Y}$ provide consistent estimators of the marginal parameters $\lambda_{1}$ and $\lambda_{2}$, respectively, given by

$${\hat{\lambda }}_{1}=1/\bar{X}\;\mathrm{and}\;{\hat{\lambda }}_{2}=1/\bar{Y}.$$

The dependence parameter θ will be estimated using the expression of the covariance $h_{\lambda_{1},\lambda_{2}}\left(\theta \right)=\mathrm{cov}\left(X,Y\right)$ described by (13). In fact, a consistent estimator of θ can be determined from the next equation

$$h_{{\hat{\lambda }}_{1},{\hat{\lambda }}_{2}}\left(\theta \right)=\frac{1}{{\hat{\lambda }}_{1}{\hat{\lambda }}_{2}{\left(1-\theta \right)}^{2}}\left\{B\left(\frac{1}{\theta },\frac{1}{\theta }\right)-\theta^{2}\right\}=S_{12}.$$

Since the function $h_{{\hat{\lambda }}_{1},{\hat{\lambda }}_{2}}\left(\theta \right)$ is strictly decreasing in terms of θ∈[0,1], the desired estimator is uniquely determined by

$$\hat{\theta }=h_{{\hat{\lambda }}_{1},{\hat{\lambda }}_{2}}^{-1}\left(S_{12}\right).$$

Observe that the lower bound of $h_{{\hat{\lambda }}_{1},{\hat{\lambda }}_{2}}\left(\theta \right)$ is ${\left({\hat{\lambda }}_{1}{\hat{\lambda }}_{2}\right)}^{-1}\left(1-\pi^{2}/6\right)$. Therefore, if the sample covariance $S_{12}$ is smaller than this lower bound, then $\hat{\theta }=1$. In addition, if $S_{12}>0$, then $\hat{\theta }=0$.

Furthermore, the asymptotic behavior of the estimator of θ can be derived from the asymptotic law of $S_{12}$, as stated below.

**Proposition** **7.** *For all θ∈(0,1), one has*

$$\sqrt{n}\left(\hat{\theta }-\theta \right)⇝N\left(0,{\left\{h_{\lambda_{1},\lambda_{2}}^{\prime }\left(\theta \right)\right\}}^{-2}\sigma^{2}\left(\theta ,\lambda_{1},\lambda_{2}\right)\right),$$

*with $\sigma^{2}\left(\theta ,\lambda_{1},\lambda_{2}\right)=\mathrm{var}\left\{\left(X-\frac{1}{\lambda_{1}}\right)\left(Y-\frac{1}{\lambda_{2}}\right)\right\}$.*

**Proof.**  In fact, it is well known (see, e.g., Theorem 8 on p. 52 of [14]) that $S_{12}$ is a consistent and asymptotic Gaussian estimator of population covariance $h_{\lambda_{1},\lambda_{2}}\left(\theta \right)$, namely

$$\sqrt{n}\left(S_{12}-h_{\lambda_{1},\lambda_{2}}\left(\theta \right)\right)⇝N\left(0,\sigma^{2}\left(\theta ,\lambda_{1},\lambda_{2}\right)\right),$$

where $\sigma^{2}\left(\theta ,\lambda_{1},\lambda_{2}\right)=\mathrm{var}\left\{\left(X-\frac{1}{\lambda_{1}}\right)\left(Y-\frac{1}{\lambda_{2}}\right)\right\}$. The result is then derived from the Delta method and Slutsky’s lemma applied to $\hat{\theta }=h_{\lambda_{1},\lambda_{2}}^{-1}\left(S_{12}\right)$. □

The previous proposition will be useful to establish a confidence interval for θ. To this end, let us first compute

(19) $$\begin{array}{ccc} \sigma^{2}\left(\theta ,\lambda_{1},\lambda_{2}\right) & = & \mathrm{var}\{\left(X-\lambda_{1}^{-1}\right)\left(Y-\lambda_{2}^{-1}\right)\}\\ & = & E\left\{{\left(X-\lambda_{1}^{-1}\right)}^{2}{\left(Y-\lambda_{2}^{-1}\right)}^{2}\right\}-{\mathrm{cov}}^{2}\left(X,Y\right)\\ & = & E\left(X^{2}Y^{2}\right)-\frac{2}{\lambda_{2}}\;E\left(X^{2}Y\right)-\frac{2}{\lambda_{1}}\;E\left(XY^{2}\right)+\frac{4}{\lambda_{1}\lambda_{2}}E\left(XY\right)\\ & & +\frac{1}{\lambda_{1}^{2}\lambda_{2}^{2}}-{\mathrm{cov}}^{2}\left(X,Y\right). \end{array}$$

The mixed moments $E(X^{i}Y^{j})$, i,j=1,2 involved in the previous formula can be obtained from Proposition 4 as follows

(20) $$\begin{array}{c} E\left(X^{2}Y\right)=\frac{2\theta^{2}-6\theta +2}{\lambda_{1}^{2}\lambda_{2}{\left(1-\theta \right)}^{3}}\\ \;-\frac{2}{\lambda_{1}^{2}\lambda_{2}}\left\{\frac{1}{\theta }B\left(\frac{1}{\theta }-1,\frac{1}{\theta }+1\right)-\frac{1}{\theta^{2}}B_{0,1}\left(\frac{1}{\theta }-1,\frac{1}{\theta }+1\right)\right\}\\ \;+\frac{2}{\lambda_{1}^{2}\lambda_{2}{\left(1-\theta \right)}^{2}}\left\{\frac{1}{\theta }B\left(\frac{1}{\theta }-1,\frac{1}{\theta }\right)-\frac{1-\theta }{\theta^{2}}B_{0,1}\left(\frac{1}{\theta }-1,\frac{1}{\theta }\right)\right\}\\ \;+\frac{2}{\lambda_{1}^{2}\lambda_{2}\theta^{2}}B_{1,0}\left(\frac{1}{\theta }-1,\frac{1}{\theta }+1\right), \end{array}$$

(21) $$\begin{array}{c} E\left(XY^{2}\right)=\frac{2\theta^{2}-6\theta +2}{\lambda_{1}\lambda_{2}^{2}{\left(1-\theta \right)}^{3}}\\ \;-\frac{2}{\lambda_{1}\lambda_{2}^{2}}\left\{\frac{1}{\theta }B\left(\frac{1}{\theta }-1,\frac{1}{\theta }+1\right)-\frac{1}{\theta^{2}}B_{0,1}\left(\frac{1}{\theta }-1,\frac{1}{\theta }+1\right)\right\}\\ \;+\frac{2}{\lambda_{1}\lambda_{2}^{2}\theta^{2}}B_{1,0}\left(\frac{1}{\theta }-1,\frac{1}{\theta }+1\right)-\frac{2}{\lambda_{1}\lambda_{2}^{2}\left(1-\theta \right)\theta^{2}}B_{1,0}\left(\frac{1}{\theta }-1,\frac{1}{\theta }\right) \end{array}$$

and

(22) $$\begin{array}{c} E\left(X^{2}Y^{2}\right)=\frac{8\theta^{2}-16\theta +4}{\lambda_{1}^{2}\lambda_{2}^{2}{\left(1-\theta \right)}^{4}}\\ \;-\frac{8}{\lambda_{1}^{2}\lambda_{2}^{2}}\left\{-\frac{1}{\theta^{2}}B_{1,0}\left(\frac{1}{\theta }-1,\frac{1}{\theta }+1\right)+\frac{1}{\theta^{3}}B_{1,1}\left(\frac{1}{\theta }-1,\frac{1}{\theta }+1\right)\right\}\\ \;+\frac{4}{\lambda_{1}^{2}\lambda_{2}^{2}{\left(1-\theta \right)}^{2}}\left\{-\frac{1}{\theta^{2}}B_{1,0}\left(\frac{1}{\theta }-1,\frac{1}{\theta }\right)+\frac{1-\theta }{\theta^{3}}B_{1,1}\left(\frac{1}{\theta }-1,\frac{1}{\theta }\right)\right\}. \end{array}$$

Using the properties of the partial derivatives of the beta function (see Appendix A) and putting (11), (13), (19)–(22) and together, it follows that

(23) $$\sigma^{2}\left(\theta ,\lambda_{1},\lambda_{2}\right)=a_{1}B^{2}\left(\frac{1}{\theta },\frac{1}{\theta }\right)+a_{2}B\left(\frac{1}{\theta },\frac{1}{\theta }\right)+a_{3},$$

where

- $a_{1}=-\frac{1}{\lambda_{1}^{2}\lambda_{2}^{2}{\left(1-\theta \right)}^{4}}$,
- $\begin{array}{ll} a_{2}= & \frac{-4{\left(1-\theta \right)}^{2}\psi^{{}^{\prime }}\left(\frac{2}{\theta }\right)+4{\left(\left(1-\theta \right)\psi \left(\frac{2}{\theta }\right)-\left(1-\theta \right)\psi \left(\frac{1}{\theta }\right)+\theta^{2}\right)}^{2}}{\lambda_{1}^{2}\lambda_{2}^{2}{\left(1-\theta \right)}^{4}\theta^{2}}\\ & +\frac{2\theta^{2}}{\lambda_{1}^{2}\lambda_{2}^{2}{\left(1-\theta \right)}^{4}} \end{array}$,
- $a_{3}=\frac{-4\theta^{3}+2\theta^{2}-4\theta +1}{{\left(1-\theta \right)}^{4}\lambda_{1}^{2}\lambda_{2}^{2}}$,

with $\psi^{{}^{\prime }}\left(x\right)$ is the derivative of the Digamma function ψ(x).

From Proposition 7, one can build an asymptotic confidence interval for the dependence parameter θ. Thus, the (1−α)×100% confidence interval for θ is given by the following formula:

$$\hat{\theta }\pm z_{\alpha /2}\frac{h^{\prime }{\left(\hat{\theta }\right)}^{-1}\sigma \left(\hat{\theta },{\hat{\lambda }}_{1},{\hat{\lambda }}_{2}\right)}{\sqrt{n}}.$$

## 5. Simulation Study

In this section, we will illustrate the performance of $\hat{\theta }$, the estimator of the dependence parameter θ. We will be examining the finite-sample accuracy of our estimates for different sample sizes. An asymptotic confidence interval for θ will also be provided.

More specifically, let $\left(X_{11},X_{12}\right),\cdots ,\left(X_{n1},X_{n2}\right)$ be mutually independent copies of the random vector $\left(X_{1},X_{2}\right)$∼$BED^{-}\left(\theta ,\Lambda \right)$. Consistent estimators of $\lambda_{1}$, $\lambda_{2}$ and θ can be obtained by the method of moments in accordance with the previous section.

To assess the performance of the moment-based estimators, the marginal parameters were held fixed at Λ=(2,4), and various values of θ were chosen to cover a broader range of negative dependence. Different sample sizes, *n*, are considered, and each scenario was replicated 500 times. As estimation of the parameters $\lambda_{1}$ and $\lambda_{2}$ is standard, we focus on the results of estimating θ.

Recall that the method of moments, used to estimate the model parameters $\lambda_{1}$ and $\lambda_{2}$, requires estimation of the dependence parameter θ∈(0,1) by solving for the unique root of

$$h_{{\hat{\lambda }}_{1},{\hat{\lambda }}_{2}}\left(\theta \right)=\frac{1}{{\hat{\lambda }}_{1}{\hat{\lambda }}_{2}{\left(1-\theta \right)}^{2}}\left\{B\left(\frac{1}{\theta },\frac{1}{\theta }\right)-\theta^{2}\right\}=S_{12}.$$

Table 1 exhibits the estimate $\hat{\theta }$ of the dependence parameter θ, bias, mean squared error (MSE), and confidence interval estimations for θ. It illustrates that simulation results obtained by the method of moments are consistent. In fact, $\hat{\theta }$ provides a good estimator for the dependence parameter θ, bias, and MSE of $\hat{\theta }$ decrease as the sample size increases. As expected, the confidence intervals get narrower as the sample size grows. Moreover, we observed from many more simulations, not presented here, that the estimator $\hat{\theta }$ performs very well regardless of the choice of $\lambda_{1}$ and $\lambda_{2}$.

## 6. Conclusions

The main purpose of this paper was to introduce a new class of bivariate exponential distribution that fully covers the negative dependence. The concept of counter-monotonic shock was used to create the negative dependence among the exponential components of the model. The basic features of this class were studied, and moment-based estimators of model parameters were derived. This family of distributions could be easily interpreted and simulated. Moreover, the proposed method can be adapted to derive a general model describing both negative and positive dependence. The Fréchet family of distribution can be a good candidate to construct such a model. In this direction, provided exponential marginal distributions *F* and *G*, this family of distributions is defined, for any $\theta_{0}\in \left[0,1\right]$, by

$$H_{F}\left(x,y\right)=\theta_{0}M\left(x,y\right)+\left(1-\theta_{0}\right)W\left(x,y\right),$$

where

$$M\left(x,y\right)=\min\left[F(x),G(y)\right]$$

and

$$W\left(x,y\right)=\sup\left[F(x)+G(y)-1,0\right].$$

Let us consider independent random variables *U*, $Y_{1}$, $Y_{2}$, and *Z* such that *U*∼$U_{\left[0,1\right]}$, $Y_{i}$∼Exp($\lambda_{i}\left(1-\theta \right)$), i=1,2 and *Z*∼Bernoulli$\left(\theta_{0}\right)$ with $\left(\theta_{0},\theta \right)\in {\left[0,1\right]}^{2}$. The key idea allowing to build such a model is based on the fact that

$$\left(G_{1}^{-1}\left(U\right),ZG_{2}^{-1}\left(U\right)+\left(1-Z\right)G_{2}^{-1}\left(1-U\right)\right)∼H_{F}.$$

Indeed, one can construct a bivariate exponential random variable by

$$\begin{array}{ccc} X & = & \min\left\{Y_{1},G_{1}^{-1}\left(U\right)\right\},\\ Y & = & \min\left\{Y_{2},ZG_{2}^{-1}\left(U\right)+\left(1-Z\right)G_{2}^{-1}\left(1-U\right)\right\}. \end{array}$$

It can be verified that the marginal distributions of (X,Y) are fixed, namely, *X*∼$\mathrm{Exp}(\lambda_{1})$ and *Y*∼$\mathrm{Exp}(\lambda_{2})$. This general model will be explored in a forthcoming paper.

## Figures and Tables

**Table 1:** Moment-based estimation for θ.

	*n*	$\hat{\theta }$	Bias ($\hat{\theta }$)	MSE ($\hat{\theta }$)	95% C.I
θ=0.2	50	0.2544	0.0544	0.0123	(0.0009,0.5215)
	100	0.2291	0.0291	0.0071	(0.0212,0.4371)
	200	0.2056	0.0056	0.0040	(0.0378,0.3735)
	300	0.2012	0.0012	0.0039	(0.0602,0.3423)
	400	0.2009	0.0009	0.0031	(0.0785,0.3233)
	500	0.1992	−0.0007	0.0027	(0.0885,0.3111)
	1000	0.1994	−0.0006	0.0016	(0.1211,0.2776)
θ=0.4	50	0.3921	−0.0079	0.0097	(0.1510,0.6331)
	100	0.3978	−0.0022	0.0051	(0.2263,0.5693)
	200	0.3982	−0.0018	0.0022	(0.2769,0.5195)
	300	0.3987	−0.0013	0.0014	(0.2995,0.4978)
	400	0.3991	−0.0009	0.0012	(0.3133,0.4850)
	500	0.3995	−0.0004	0.0004	(0.3211,0.4779)
	1000	0.4003	0.0003	0.0004	(0.3460,0.4547)
θ=0.5	50	0.4967	−0.0032	0.0095	(0.2152,0.7782)
	100	0.5014	0.0014	0.0053	(0.3007,0.7021)
	200	0.5010	0.0009	0.0024	(0.3592,0.6427)
	300	0.5007	0.0007	0.0015	(0.3850,0.6165)
	400	0.4994	0.0005	0.0011	(0.3994,0.5994)
	500	0.4995	−0.0004	0.0010	(0.4101,0.5889)
	1000	0.5002	0.0002	0.0004	(0.4369,0.5635)
θ=0.6	50	0.5976	−0.0024	0.0113	(0.2594,0.9357)
	100	0.6018	0.0019	0.0055	(0.3608,0.8428)
	200	0.5984	−0.0016	0.0024	(0.4290,0.7677)
	300	0.6009	0.0009	0.0020	(0.4620,0.7398)
	400	0.6007	0.0007	0.0012	(0.4798,0.7215)
	500	0.6004	0.0005	0.0011	(0.4930,0.7080)
	1000	0.5999	−0.0001	0.0005	(0.5240,0.6757)
θ=0.8	50	0.7912	−0.0087	0.01249	(0.3130,1.2500)
	100	0.7983	−0.0016	0.0068	(0.4576,1.1390)
	200	0.8008	0.0008	0.0039	(0.5589,1.0427)
	300	0.7992	−0.0007	0.0026	(0.6022,0.9962)
	400	0.8007	0.0007	0.0019	(0.6297,0.9718)
	500	0.8004	0.0004	0.0015	(0.6475,0.9533)
	1000	0.8002	0.0002	0.0008	(0.6921,0.9083)

## Data Availability

Not applicable.

## Appendix A

Recall the beta function defined for x>0, y>0

$$B\left(x,y\right)=\int_{0}^{1}t^{x-1}{\left(1-t\right)}^{y-1}dt.$$

Furthermore, the partial derivatives of the beta function are given, for x,y>0, by

$$B_{p,q}\left(x,y\right)=\frac{\partial^{p+q}B}{\partial x^{p}\partial y^{q}}\left(x,y\right)=\int_{0}^{1}t^{x-1}{\left(1-t\right)}^{y-1}\ln^{p}\left(t\right)\ln^{q}\left(1-t\right)dt.$$

The application of some algebra yields

- $B_{0,1}\left(\frac{1}{\theta }-1,\frac{1}{\theta }\right)=\frac{\theta }{1-\theta }B\left(\frac{1}{\theta },\frac{1}{\theta }\right)+\frac{2-\theta }{1-\theta }B_{0,1}\left(\frac{1}{\theta },\frac{1}{\theta }\right)$.
- $B_{0,1}\left(\frac{1}{\theta }-1,\frac{1}{\theta }+1\right)=\frac{\theta }{1-\theta }B\left(\frac{1}{\theta },\frac{1}{\theta }\right)+\frac{1}{1-\theta }B_{0,1}\left(\frac{1}{\theta },\frac{1}{\theta }\right)$.
- $B_{1,0}\left(\frac{1}{\theta }-1,\frac{1}{\theta }\right)=-\frac{\theta }{{\left(1-\theta \right)}^{2}}B\left(\frac{1}{\theta },\frac{1}{\theta }\right)+\frac{2-\theta }{1-\theta }B_{1,0}\left(\frac{1}{\theta },\frac{1}{\theta }\right)$.
- $B_{1,0}\left(\frac{1}{\theta }-1,\frac{1}{\theta }+1\right)=-\frac{\theta }{{\left(1-\theta \right)}^{2}}B\left(\frac{1}{\theta },\frac{1}{\theta }\right)+\frac{1}{1-\theta }B_{1,0}\left(\frac{1}{\theta },\frac{1}{\theta }\right)$.
- $\begin{array}{ll} B_{1,1}\left(\frac{1}{\theta }-1,\frac{1}{\theta }\right)= & -\frac{\theta^{2}}{{\left(1-\theta \right)}^{2}}B\left(\frac{1}{\theta },\frac{1}{\theta }\right)+\frac{\theta }{1-\theta }B_{1,0}\left(\frac{1}{\theta },\frac{1}{\theta }\right)\\ & -\frac{\theta }{{\left(1-\theta \right)}^{2}}B_{0,1}\left(\frac{1}{\theta },\frac{1}{\theta }\right)+\frac{2-\theta }{1-\theta }B_{1,1}\left(\frac{1}{\theta },\frac{1}{\theta }\right) \end{array}$
- $\begin{array}{ll} B_{1,1}\left(\frac{1}{\theta }-1,\frac{1}{\theta }+1\right)= & -\frac{\theta^{2}}{{\left(1-\theta \right)}^{2}}B\left(\frac{1}{\theta },\frac{1}{\theta }\right)+\frac{\theta }{1-\theta }B_{1,0}\left(\frac{1}{\theta },\frac{1}{\theta }\right)\\ & -\frac{\theta }{{\left(1-\theta \right)}^{2}}B_{0,1}\left(\frac{1}{\theta },\frac{1}{\theta }\right)+\frac{1}{1-\theta }B_{1,1}\left(\frac{1}{\theta },\frac{1}{\theta }\right) \end{array}$
- $B_{0,1}\left(\frac{1}{\theta },\frac{1}{\theta }\right)=B_{1,0}\left(\frac{1}{\theta },\frac{1}{\theta }\right)=B\left(\frac{1}{\theta },\frac{1}{\theta }\right)\left\{\psi \left(\frac{1}{\theta }\right)-\psi \left(\frac{2}{\theta }\right)\right\}$.
- $B_{1,1}\left(\frac{1}{\theta },\frac{1}{\theta }\right)=B\left(\frac{1}{\theta },\frac{1}{\theta }\right)\left\{{\left(\psi \left(\frac{1}{\theta }\right)-\psi \left(\frac{2}{\theta }\right)\right)}^{2}+\psi^{{}^{\prime }}\left(\frac{2}{\theta }\right)\right\}$,

where $\psi^{{}^{\prime }}\left(x\right)$ is the derivative of the Digamma function ψ(x).
